# Anti-inflammatory response to curcumin supplementation in chronic kidney disease and hemodialysis patients: A systematic review and meta-analysis

**DOI:** 10.22038/AJP.2022.20049

**Published:** 2022

**Authors:** Elham Emami, Saeid Heidari-Soureshjani, Catherine MT Sherwin

**Affiliations:** 1 *Department of Pediatric Nephrology, Emam Hossein Educational Hospital, School of Medicine, Shahid Beheshti University of Medical Sciences, Tehran, Iran*; 2 *Modeling in Health Research Center, Shahrekord University of Medical Sciences, Shahrekord, Iran*; 3 *Department of Pediatric Clinical Pharmacology, Department of Pharmacology & Toxicology, Wright State University Boonshoft School of Medicine, Dayton Children's Hospital, One Children's Plaza, Dayton, Ohio, USA*

**Keywords:** Chronic kidney disease, Hemodialysis, Curcumin, Inflammation

## Abstract

**Objective:**

This study was designed to determine the association curcumin has on pro-inflammatory biomarkers in patients with chronic kidney disease (CKD (and in those receiving hemodialysis (HD).

**Materials and Methods:**

This meta-analysis was undertaken following PRISMA guidelines. An extensive systematic review was undertaken until 10/11/2021 using PubMed, Web of Science (ISI), and Scopus databases. The standardized mean difference (SMD) and 95% confidence intervals (CI) were used to estimate the overall effect size of curcumin on serum high-sensitivity C-reactive protein (hs-CRP), and pro-inflammatory cytokines including interleukin 6 (IL-6), and tumor necrosis factor α (TNF-α) in patients with CKD and those receiving HD.

**Results:**

Overall, ten randomized controlled trials (RCTs) comprising 523 patients were incorporated into the systematic review and meta-analysis. The results showed that when compared with control groups, there was no significant effect observed linking curcumin and IL-6 (SMD = 0.24%, 95% CI = -0.14 to 0.62, p = 0.221), TNF-α (SMD = 0.11%, 95% CI = -0.19 to 0.40, p = 0.480) or hs-CRP (SMD = -0.17%, 95% CI = -0.36 to 0.03, p = 0.093). The analysis determined no publication bias related to the influence of curcumin on IL-6, TNF-α or acute phase reactant, hs-CRP. The Egger’s and Begg’s test results were not statistically significant (p˃0.20).

**Conclusion:**

In patients with CKD and those receiving HD, the use of curcumin supplementation has no statistically significant effect on the anti-inflammatory biomarkers reviewed in this study.

## Introduction

Chronic kidney disease (CKD) is unrivaled as one of the most significant health challenges worldwide that contribute to overall morbidity and mortality. CKD is an irreversible and progressive disease considered a significant risk factor for many life-threatening diseases. This includes cardiovascular disease (disorders of the heart and blood vessels) and mineral and bone disorders (Ammirati, 2020; Collaboration, 2020; Hasan et al*.*, 2018; Miller, 2014). Patients are generally offered different treatment options following CKD development, such as hemodialysis (HD). CKD leads to social impairment depressive symptoms and can be a substantial financial burden. Patients' other complications and issues include pain, weakness after dialysis, anemia, and recurring infections. Low medication adherence can worsen a patient’s condition and considerably reduce his/her quality of life (Bartaula et al*.*, 2019; Moreno Velásquez et al*.*, 2019; Pretto et al*.*, 2020). 

Given the overall morbidity and mortality associated with CKD and the significant impact on affected patients, this disease deserves far more awareness within health policy decision-making. This is because it is largely preventable, and effective treatments are available that improve patients' quality of life (Corsonello et al*.*, 2020). The choice of safe and effective medications for this disease involves determining various aspects such as kidney function and monitoring for declines, as this induces changes in drug pharmacokinetics and subsequent drug effectiveness. Appropriate administration of drugs when managing other comorbid conditions and uremic complications is essential (Whittaker et al*.*, 2018). This is because some medications induce drug-drug interactions, and some procedures result in undesirable or harmful effects that increase the consequences of the potential deterioration of the disease resulting in hospital admission and extended lengths of hospital stays (Subeesh et al*.*, 2020). It is recommended to use disease-appropriate, safe, effective treatments and complementary therapies.

Complementary and alternative medicines are becoming increasingly popular worldwide, primarily herbal medicines also called phytotherapy (Welz et al*.*, 2018; Yuan et al*.*, 2016). Among these is curcumin, a yellow phytochemical produced by *Curcuma longa,* a member of the ginger family (Zingiberaceae). It has been reported to have a beneficial effect on renal diseases and to have anti-inflammatory and antioxidant properties (L. Alvarenga et al., 2020; Bagherniya et al*.*, 2021; Mohammad pour et al., 2019; Najafi et al*.*, 2015). However, there is a knowledge gap, and there are still ambiguous aspects to reports related to the use of this herb. Some studies have shown that it can downregulate the pro-inflammatory cytokines tumor necrosis factor α (TNF-α) and interleukin-1 (IL-1) but that it does not affect interleukin 6 (IL-6) and interleukin-8 (IL-8) (Gorabi et al*.*, 2021). Other studies have reported that curcumin does not reduce inflammation or oxidative stress markers (Rodrigues et al*.*, 2021). 

In patients with CKD and those receiving HD, their immune system response activity is disrupted and this leads to pro-inflammatory cytokine dysregulation and a state of persistent inflammation. This is associated with complications, including atherosclerotic vascular disease, protein-energy wasting, depression, and osteoporosis (Carrero et al*.*, 2008; Tinti et al*.*, 2021). Based on the global prevalence of CKD and the persistent need to improve treatment, this meta-analysis systematically investigates the consequence of curcumin supplementation on pro-inflammatory constituents to improve the patients’ conditions with CKD.

## Materials and Methods

**Data sources and search strategy**

The present systematic review and meta-analysis were executed using PRISMA guidelines (http://prisma-statement.org/prismastatement/Checklist.aspx). An extensive systematic review was undertaken until 10/11/2021 using Web of Science (ISI), PubMed, and Scopus databases. These central and MeSH keywords were used in the search: ((“chronic kidney diseases” OR “chronic kidney insufficiency” OR “chronic renal diseases” OR “chronic renal insufficiency” OR *dialysis* OR “hemodialysis” OR “renal dialysis” OR “extracorporeal dialysis”) AND (“curcumin” OR “curcuma” OR Curcuma longa” OR “turmeric AND ( “anti-inflammatory” OR “inflammation” OR *inflammatory cytokine* OR CRP OR “c-reactive protein” OR “hs-CRP” OR “interleukin-1” OR “interleukin-6” OR “IL-1” OR tumor necrosis factor OR TNF)).

**Study selection**

**Figure 1:**
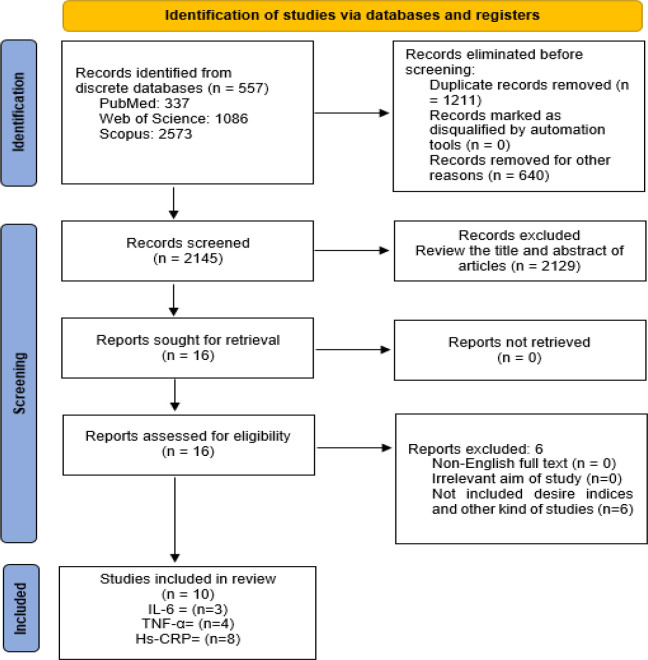
Flowchart for including studies in the meta-analysis

Available peer-reviewed publications were imported into EndNote X8 (8 November 2016, Thomson Reuters) to detect and remove identical publications. Two investigators objectively screened the titles and abstracts of the reported research established by the inclusion and exclusion criteria. Based on inclusion criteria, clinical trials that addressed the anti-inflammatory response associated with curcumin supplementation in patients with CKD receiving HD were included in the systematic review and meta-analysis. Articles not published in English were among the study's exclusion criteria. Publications in the format of letters to the editor, case reports, narrative reviews, and studies involving animals were excluded from the study. The full texts of all eligible publications were independently reviewed. A consensus was achieved for any potential disagreement during the review through discussion with a third team member. The flowchart used for the examination strategy is illustrated in Figure 1. 

**Data extraction and quality assessment**

Two researchers extracted the data objectively, and any inconsistencies were resolved by discussion. For each publication and study included, the following data were extracted: the first author's details, year of publication, country of study origin, mean age of study population, sample size, length/time of follow-up, curcumin dosage (mg/day), duration of intervention, and the mean and standard deviation (±SD) level of the pro-inflammatory biomarkers before and following the intervention.

**Statistical analysis**

The standardized mean difference (SMD) and 95% confidence intervals (CI) were used to estimate the overall effect size of curcumin on serum high-sensitivity C-reactive protein (hs-CRP), interleukin 6 (IL-6), TNF-α in patients with CKD receiving HD. When studies did not report the ±SD of the mean difference, this was calculated by SD^2^ = [(SD baseline^2^ + SD final^2^) - (2 × 0.8 × SD baseline × SD final)]. The heterogeneity of studies was tested using Cochran’s Q test (reported by chi-square, with p<0.1 considered significant) and by estimating the statistic I^2^. Begg's and Egger's tests were used to estimate potential publication and accumulation bias. All statistical analyses were accomplished using Stata 14.0 (Stata LLC, College Station, TX, USA), with p<0.05 judged statistically significant.

## Results

**Search results and characteristics of selected studies**

The PRISMA flowchart is illustrated in Figure 1. The initial electronic search retrieved 557 titles/abstracts. From the total articles retrieved, 264 articles were removed due to duplicate titles. Some other titles/abstracts were also excluded (n= 6) as they did not match our study aims: (Jiménez-Osorio et al., 2016 ▶; Pakfetrat et al., 2015 ▶; Salarolli et al., 2021 ▶; Shelmadine et al., 2017 ▶; Vanaie et al., 2019 ▶; Yang et al., 2015 ▶). In total, ten clinical trials (which involved 523 patients) were incorporated into the meta-analysis (L. Alvarenga et al., 2020 ▶; Kabodan et al., 2018 ▶; Khajehdehi et al., 2011 ▶; Moreillon et al., 2013 ▶; Pakfetrat et al., 2014 ▶; Rodrigues et al., 2021 ▶; Samadian et al., 2017 ▶; Shafabakhsh et al., 2020 ▶; Vafadar-Afshar et al., 2021 ▶; Vafadar Afshar et al., 2020 ▶). From the selected trials, three studies investigated IL-6 (Kabodan et al., 2018 ▶; Moreillon et al., 2013 ▶; Vafadar-Afshar et al., 2021 ▶), eight studies involved hs-CRP (L. Alvarenga et al., 2020 ▶; Kabodan et al., 2018 ▶; Moreillon et al., 2013 ▶; Pakfetrat et al., 2014 ▶; Rodrigues et al., 2021 ▶; Samadian et al., 2017 ▶; Shafabakhsh et al., 2020 ▶; Vafadar Afshar et al., 2020 ▶) and four studies analyzed TNF-α (Khajehdehi et al., 2011 ▶; Moreillon et al., 2013 ▶; Pakfetrat et al., 2014 ▶; Vafadar-Afshar et al., 2021 ▶) (Figure 1). 

All studies incorporated into this study were published between 2013 and 2021. Detailed data from the studies, including sample size, length/time of follow-up, number of participants in the intervention and control groups, study design, curcumin dose and duration of administration, year of study, and location of the study are summarized in Table 1.

**The outcome of the meta-analyses of the effect of curcumin on serum level of IL-6**

**Figure 2:**
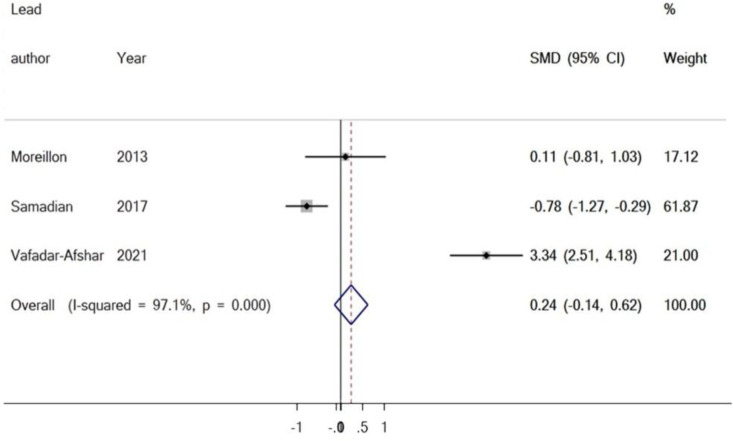
Forest plot presenting SMD and 95% CI for the impact of curcumin on IL-6

**Figure 3:**
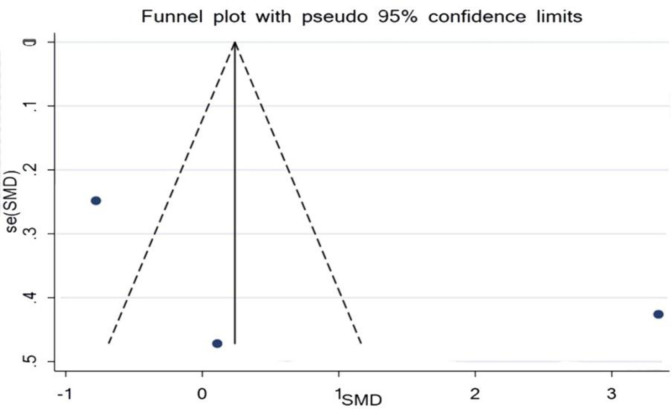
Evaluation of publication bias in the included studies for the effect of curcumin on IL-6

Three studies examined the influence of curcumin and IL-6, with 71 participants in the intervention group and 70 participants in the control group. The results revealed there was no significant increase in the serum level of IL-6 when matched against the control group (SMD = 0.24%, 95% CI = -0.14 to 0.62, p=0.221) (Figure 2). Analysis of the study, investigating the impact of curcumin on IL-6, determined there was publication bias. The conclusion of Egger’s test was equal to (p =0.508), and Begg’s test (p =0.602) produced no statistically significant results (Figure 3). 

**Table 1:** Characteristics of the studies considered for reviewing the anti-inflammatory relationship response to curcumin supplementation in CKD and HD patients

**Study author**	**Year**	**Country**	**Gender**	**Age mean**	**N**	**Renal disorder**	**Total dose (per day)**	**Follow up**	**Intervention group mean±SD**	**Control group mean±SD**
**Interleukin 6 (IL-6)**										
Moreillon (Moreillon et al., 2013 ▶)	2013	USA	NA*	56	16	CKD**	824 mg	8 weeks	35.5±46.7	30.7±46.7
Samadian (Samadian et al., 2017 ▶)	2017	Iran	NA	49	71	HD***	1500 mg	12 weeks	5.73±5.16	35.37±53.59
Vafadar-Afshar (Vafadar-Afshar et al., 2021 ▶)	2021	Iran	62.97% male 37.03% female	57	54	HD	120 mg	12 weeks	34.4±4.8	21.6±2.51
**Tumor necrosis factor α (TNF-α)**										
Khajehdehi (Khajehdehi et al., 2011 ▶)	2011	Iran	55% male and 45% female	52	40	CKD	1500 mg	2 months	12.8±2.9	18.4±24.1
Moreillon (Moreillon et al., 2013 ▶)	2013	USA	NA	56	16	CKD	824 mg	8 weeks	31.4±43.5	34.4±40.5
Pakfetrat (Pakfetrat et al., 2014 ▶)	2014	Iran	60% male and 40% female	53	100	HD	1500 mg	8 weeks	36.36±42.38	84.11±65.14
Vafadar-Afshar (Vafadar-Afshar et al., 2021 ▶)	2021	Iran	62.97% male 37.03% female	57	54	HD	120 mg	12 weeks	43.4±6.79	27.8±3.05
**High-sensitivity C-reactive protein (hs-CRP)**										
Moreillon (Moreillon et al., 2013 ▶)	2013	USA	NA	56	16	CKD	824 mg	8 weeks	2±1	3±2
Pakfetrat (Pakfetrat et al., 2014 ▶)	2014	Iran	60% male and 40% female	53	100	HD	1500 mg	8 weeks	10.8±9.7	7±8.9
Samadian (Samadian et al., 2017 ▶)	2017	Iran	NA	49	71	HD	1500 mg	12 weeks	1.23±82.3	53±4.2
Kabodan (Kabodan et al., 2018 ▶)	2018	Iran	NA	NA	64	HD	800 mg	8 weeks	22.1±37	19±26.76
Alvarenga (L. Alvarenga et al., 2020 ▶)	2020	Brazil	NA	NA	28	HD	2500 mg	12 weeks	3.8±2.15	2±1.35
Shafabakhsh (Shafabakhsh et al., 2020 ▶)	2020	Iran	60.4% male 39.6% female	57	53	HD	80 mg	12 weeks	5.4±2.5	4.5±2
Vafadar-Afshar (Vafadar Afshar et al., 2020 ▶)	2020	Iran	62.97% male 37.03% female	57	54	HD	120 mg	12 weeks	13±8.5	6.9±3.58
Rodrigues (Rodrigues et al., 2021 ▶)	2021	Brazil	67.45% male 32.55% female	53	43	HD	1000 mg	12 weeks	5.2±8.5	5.6±8.5

* NA; Not applicable, **CKD; Chronic kidney disease, ***HD; Hemodialysis

**Results from the meta-analyses of curcumin’s effect on serum TNF-α**

**Figure 4:**
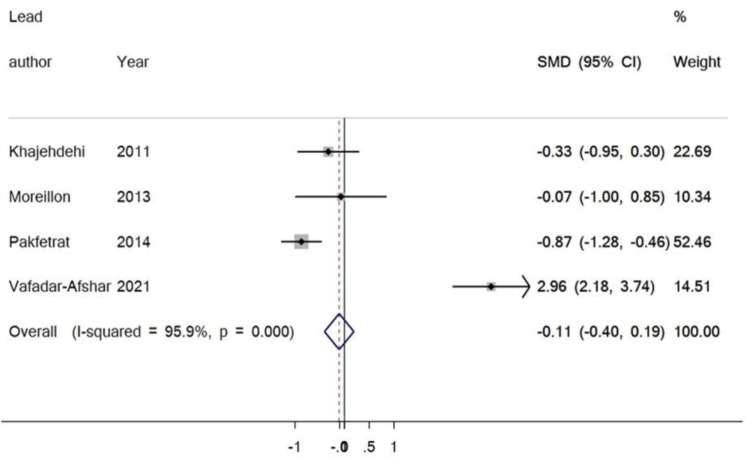
Forest plot representing the SMD and 95% CI for the impact of curcumin on TNF-α

**Figure 5:**
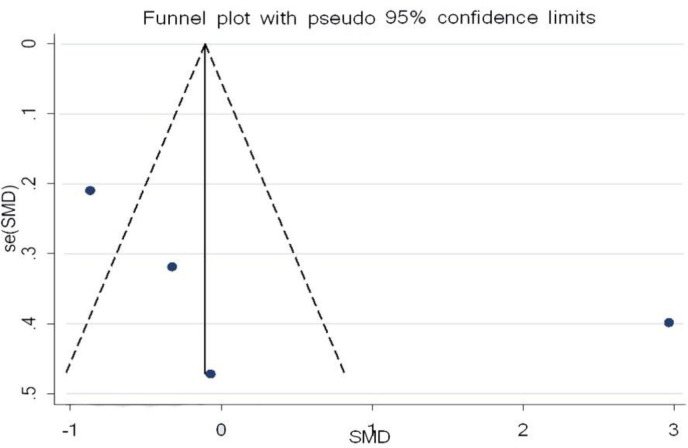
Evaluation of publication bias in the included studies for the consequence of curcumin on TNF-α

In this meta-analysis, four studies investigated the influence of curcumin supplementation on TNF-α. There were 106 participants within the intervention group and 104 participants in the control group. The findings exhibited no significant change in the serum level of TNF-α matched to the control group (SMD = 0.11%, 95% CI = -0.19 to 0.40, P = 0.480) (Figure 4). There was no publication bias. The result of Egger’s test was equal to (p = 0.342), and Begg’s test (p=0.308) produced no statistically significant results (Figure 5). 

**Outcome from meta-analyses of the effect of curcumin on serum levels of hs-CRP**

**Figure 6:**
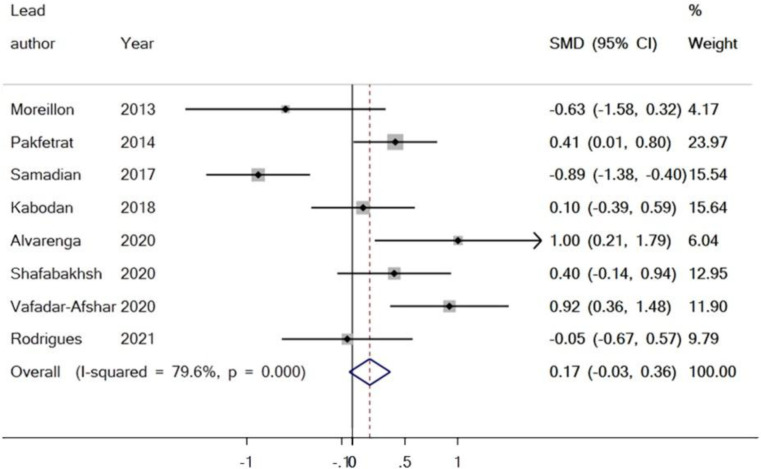
Forest plot presenting SMD and 95% CI for the outcome of curcumin on hs-CRP

**Figure 7:**
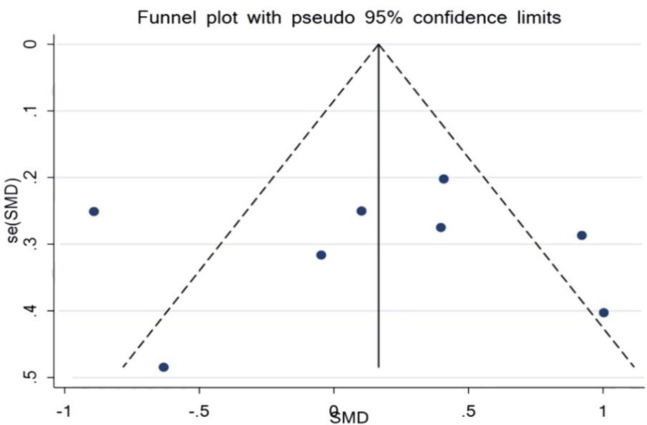
Evaluation of publication bias in the included studies for the outcome of curcumin on hs-CRP

Within this review, eight studies assessed the relationship between curcumin supplementation and the acute phase reactant, hs-CRP. There were 214 participants within the intervention group and 215 participants within the control group. The results found no significant reduction in the serum level of hs-CRP when equated to the control group (SMD = -0.17%, 95% CI = -0.36 to 0.03, p=0.093) (Figure 6). Evaluation of the trials reporting the effect of curcumin on the acute phase reactant, hs-CRP, showed no publication bias. The p-value of Egger’s test was equal to p=0.982, and Begg’s test (p=0.902) yielded no statistically significant results (Figure 7). 

## Discussion

This systematic review and meta-analysis investigated the consequence of curcumin supplementation on pro-inflammatory cytokines in patients with CKD receiving HD. Curcumin is a bright yellow chemical derived from plants in the *Curcuma longa* species that was suggested to be used as a complementary therapy in multiple diseases. It has been reported to be used safely and efficiently as an adjuvant in multiple autoimmune and inflammatory diseases such as inflammatory bowel disease, sclerosis, psoriasis, osteoarthritis, and systemic lupus erythematosus. It is outlined that the wide range of bioactive substances present in the plant has anti-inflammatory, anti-tumor, and antioxidant effects (Haroyan et al., 2018 ▶; Kotha, Luthria, 2019 ▶; Kunnumakkara et al., 2017 ▶; Marton et al., 2020 ▶; J. Sharifi-Rad et al., 2020 ▶). However, there remains debate and a lack of consensus regarding curcumin's overall safety and toxicity. There are concerns regarding dosing and administration and both should be carefully considered to achieve effective therapy and to avoid adverse effects. 

Studies have suggested that by targeting specific signalling pathways, curcumin induces anti-inﬂammatory and antioxidant effects through NF-kB and Nrf-2 modulation in patients with CKD (Rahban et al., 2020 ▶; Xu et al., 2021 ▶). Various metabolic and biochemical alterations are associated with CKD due to the systemic oxidative stress and inﬂammation the disease promotes. This progresses the patient’s disease deterioration by directly or indirectly affecting the up-regulation of NF-kB and downregulation of Nrf2. It is proposed that curcumin could be used to reduce the impact of this physiological response (Livia Alvarenga et al., 2018 ▶). Even though other studies have reviewed inflammatory factors and various acute phase reactants and cytokines in a range of diseases, this study only investigated factors in patients with CKD and HD receiving curcumin. In this study, there was no significant effect associated with curcumin on IL-6 (SMD = 0.24%, 95% CI = -0.14 to 0.62, p=0.221), TNF-α (SMD = 0.11%, 95% CI = -0.19 to 0.40, P = 0.480) and hs-CRP (SMD = -0.17%, 95% CI = -0.36 to 0.03, p=0.093). Consistent with the results of this study, White et al. reported that turmeric (from the ground up roots of the *Curcuma longa* plant) and curcumin were not linked with a decrease in several inflammatory markers including hs-CRP (MD -1.44 mg/L, 95%CI -2.94 to 0.06, p=0.06, six studies), CRP (MD -2.71 mg/L, 95%CI -5.73 to 0.31, p=0.08, five studies), IL-1 (MD -4.25 pg/ml, 95%CI -13.32 to 4.82, p=0.36, two studies), TNF α (MD -1.23 pg/ml, 95%CI -3.01 to 0.55, p=0.18, seven studies) and IL-6 (MD -0.71 pg/ml, 95%CI -1.68 to 0.25, p=0.15). These studies were undertaken in patients with chronic inflammatory diseases. In addition, no variations were reported among the interventions using turmeric or curcumin supplementation (White et al., 2019 ▶). 

A different meta-analysis examining the influence of curcumin on proinflammatory cytokines, reported results that did show a significant reduction in the serum levels of TNF-α (WMD = −1.61 pg/ml, 95% CI = −2.72, −0.51, p<0.001 and IL-1 (WMD = −2.33 pg/ml, 95% CI = −3.33 to −1.34, p<0.001), when matched to the placebo group. However, no significant association was reported concerning the use of curcumin supplementation as well as the reduced and increased levels of IL-6 (WMD = −0.33 pg/ml, 95% CI = −0.99–0.34, P = 0.33) and IL-8 (WMD = 0.52 pg/ml, 95% CI = −1.13–2.17, p=0.53), respectively) (Gorabi et al., 2021 ▶). 

Memarzia et al. suggested that *Curcuma longa* (turmeric) and curcumin improve the pro-inflammatory activities of IL-4, immunoglobulin E, transforming growth factor-beta, IL-17, and interferon-gamma levels. They outlined that these supplements also reverse impairments in disorders causing disturbances associated with the immune system through antioxidant and anti-inflammatory effects (Memarzia et al., 2021 ▶). A different meta-analysis proclaimed the beneficial anti-inflammatory effects of curcumin. Ferguson et al. showed an increase in IL-10 (0.49 pg/ml; 0.10 to 0.88) and a reduction in hs-CRP (-1.55 mg/L; -1.81 to -1.30), IL-8 (-0.54 pg/ml; -0.82 to -0.28), IL-6 (-1.69 pg/ml, -2.56 to -0.82), TNF α (-3.13 pg/ml; -4.62 to -1.64), and monocyte chemoattractant protein-1 (MCP-1/CCL2) (-2.48 pg/ml; -3.96 to -1.00) (Ferguson et al., 2021 ▶).

However, according to the results of our extensive analysis, curcumin, in general, has no direct effect on the reduction or regulation of inflammatory factors in the kidney and subsequently in CKD. We acknowledge that there are still contradictions within the published studies. The main issue is that different types of inflammatory factors have been investigated and that they do not all have the same effect on inflammatory processes associated with the disease. The variations in types of administration and the diversity within the compounds may cause conflicting results between the studies due to the use of different preparations of curcumin in the trials. For example, curcumin as a constituent within formulations has poor solubility and is poorly absorbed when in the free form in the digestive tract. Due to its rapid biotransformation to an inactive metabolite, the administration and formulation used within a study are critical. Recent advances in curcumin nano-formulations have been reported to enhance curcumin's bioavailability and result in encouraging blood levels (Stohs et al., 2020 ▶). There remains a lack of data associated with toxicity and potential adverse effects.

Nevertheless, there have been some promising effects associated with curcumin. These appear to be dosage-dependent and require robust dosage studies to be undertaken to determine effectiveness. As outlined, curcumin appears to have dose-dependent limitations due to its bioavailability. This is a crucial factor when considering potential therapeutic outcomes and how this may affect endpoints and results (Gupta et al., 2013 ▶; Javad Sharifi-Rad et al., 2020 ▶). Furthermore, curcumin dosage is a determinant that induces reactive oxygen species (ROS) generation, inflammatory cytokines, and cell apoptosis (Chan et al., 2006 ▶). The most substantial contradictory factor in the results from studies may be due to the severity of CKD and varying stages of kidney disease. Circulating concentrations of inflammatory mediators such as IL-1, IL-6, TNF-α, and hs-CRP are known to be correlated with the severity of the kidney disease (Crewe et al., 2017 ▶; Mihai et al., 2018 ▶) 

Critically, due to the worldwide prevelance of CKD and the associated morbidity and mortality, further clinical and experimental studies, including those using CKD animal models, are needed. Importantly, there should be more clinical trials to evaluate the potential of complementary therapies such as curcumin supplementation on the inﬂammation process and its associated expression of transcription factors in patients with CKD. To date, there is a dearth of comprehensive investigations that include all prospective inflammatory factors. This systematic review shows there has been a failure to push research to the limit when it comes to investigating inflammatory factors in patients with CKD. This was among the limitations of the present study and may explain the general lack of consensus when investigating anti-inflammatory responses (IL-6, TNF-α, and hs-CRP) to curcumin supplementation. Consequently, among this study's strength, the comprehensive nature and extensive review undertaken of available studies can be mentioned, to name just a few.

In patients with CKD receiving HD, the use of curcumin supplementation was found not to generate a statistically significant impact on the levels of anti-inflammatory biomarkers such as hs-CRP, IL-6, and TNF-α. Therefore, there is an urgent need for comprehensive clinical trials than ever utilizing robust methodologies to finally offer a more accurate appraisal of the effect of curcumin on inflammatory factors in patients with CKD receiving HD. This will help build consensus as to whether curcumin is a beneficial complementary therapy to use within this patient population to enhance their quality of life.

## Conflicts of interest

The authors have declared that there is no conflict of interest.
